# Next-generation sequencing for D47N mutation in Cx50 analysis associated with autosomal dominant congenital cataract in a six-generation Chinese family

**DOI:** 10.1186/s12886-017-0476-5

**Published:** 2017-05-19

**Authors:** Chao Shen, Jingbing Wang, Xiaotang Wu, Fuchao Wang, Yang Liu, Xiaoying Guo, Lina Zhang, Yanfei Cao, Xiuhua Cao, Hongxing Ma

**Affiliations:** 1 Department of Clinical Diagnosis, General Hospital of Daqing Oil Field, Daqing, Heilongjiang Province People’s Republic of China; 2 Department of Ophthalmology, General Hospital of Daqing Oil Field, Daqing, Heilongjiang Province People’s Republic of China

**Keywords:** Congenital cataract, GJA8, Whole exome sequencing, Next-generation sequencing

## Abstract

**Background:**

Congenital cataract is the most frequent cause of blindness during infancy or early childhood. To date, more than 40 loci associated with congenital cataract have been identified, including at least 26 genes on different chromosomes associated with inherited cataract. This present study aimed to identify the genetic mutation in a six-generation Chinese family affected with congenital cataract.

**Methods:**

A detailed six-generation Chinese cataract family history and clinical data of the family members were recorded. A total of 27 family members, including 14 affected and 13 unaffected individuals were recruited. Whole exome sequencing was performed to determine the disease-causing mutation. Sanger sequencing was used to confirm the results.

**Results:**

A known missense mutation, c. 139G > A (p. D47N), in *Cx50* was identified. This mutation co-segregated with all affected individuals and was not observed in the unaffected family members or in 100 unrelated controls. The homology modeling showed that the structure of the mutant protein was different with that wild-type Cx50.

**Conclusions:**

The missense mutation c.139G > A in GJA8 gene is associated with autosomal dominant congenital cataract in a six-generation Chinese family. The result of this present study provides further evidence that the p. D47N mutation in *CX50* is a hot-spot mutation.

## Background

Congenital cataract is the most frequent cause of blindness during infancy or early childhood, with an occurrence of 1–15/10,000 live births worldwide [1, 2]. It explains for 10%–30% of childhood blindness [3]. Congenital cataract is characterized by the presence of an opacification of the lens at birth or during babyhood. On the basis of morphology, congenital cataract can be classified into several subtypes, including nuclear, sutural, polar, cortical cataract, etc. [4]. Congenital cataract pathogenesis involves several distinct reasons including gene defects, chromosomal abnormalities, metabolic disorders, and infections during embryogenesis. Approximately half of congenital cataracts are inherited [3]. Though autosomal recessive and X-linked inheritances have been reported, inheritance is mainly autosomal dominant [5]. Up to date, over 40 loci associated with congenital cataract have been confirmed, including no less than 26 genes on different chromosomes related to congenital cataract [6, 7]. Among these mutant genes, the connexin genes and crystallin genes are the most widespread. Briefly, half of the mutations were discovered in the crystalline genes, such as alpha crystallins, beta crystallins and gamma crytallins, and approximately 25% involve mutations in membrane transport genes, such as connexin proteins (*Cx43*, *Cx46*, and *Cx50*) [5–14].

In current study, we utilized next-generation sequencing of whole exome to investigate genetic defects in a Chinese pedigree with congenital cataract.

## Methods

### Subject recruitment and DNA sampling

**Fig. 1:**
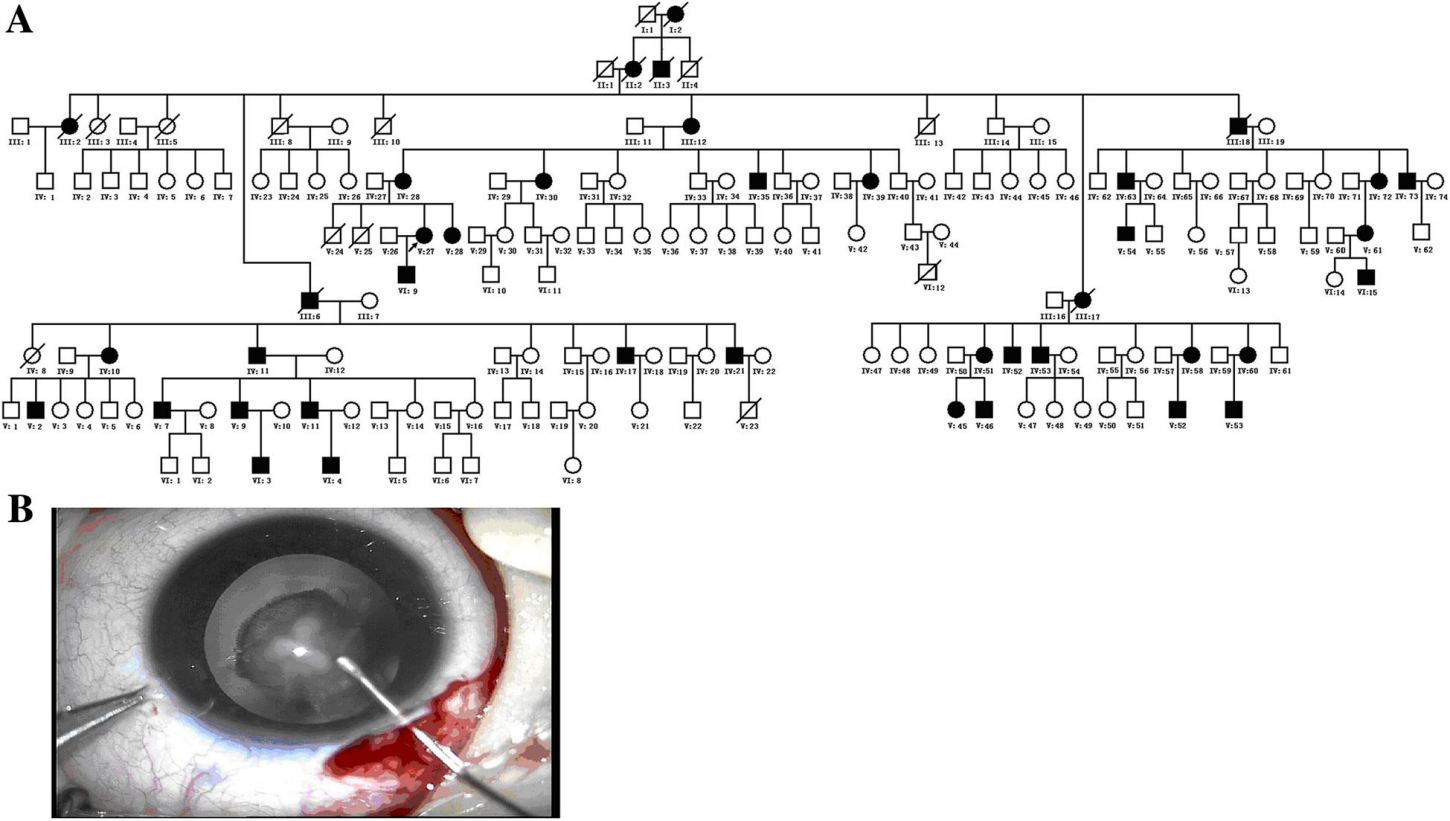
Clinical evaluation of a Chinese pedigree with autosomal dominant congenital cataract. **a** Pedigree of a six-generation Chinese family with autosomal dominant congenital cataracts. The *arrow* indicates the proband. Squares and circles symbolize males and females, respectively. *Black and white* denote the status of family members affected or unaffected, respectively, by congenital cataract. **b** Photo was taken with a surgical microscope

A six-generation Chinese cataract family was examined at the General Hospital of Daqing Oil Field, Heilongjiang province, China. Pedigree medical history was taken directly by interviewing the family members. A total of 27 family members, including 14 affected (III12, IV11, IV28, IV30, IV39, IV72, IV73, V9, V11, V27, V28, VI3, VI9, and VI15) and 13 unaffected individuals (IV40, IV68, IV69, IV70, IV71, IV74, V10, V14, V19, V42, V57, V62, VI13) were recruited (Fig. 1). Ethical approval for current research was obtained from the ethics committee of General Hospital of Daqing Oil Field and the study was conducted according to the Declaration of Helsinki of the World Medical Association. All members recruited in this study underwent ophthalmologic examinations, including slit lamp ophthalmoscopy, biometry, visual acuity, and fundus examination. In addition, 100 unrelated healthy subjects without cataracts were also recruited from General Hospital of Daqing Oil Field.

DNA samples were extracted using the QIAamp DNA Blood Midi Kit (Qiagen, Hilden, Germany) from peripheral blood.

### Exome sequencing

Ten patients (III12, IV11, IV28, IV30, IV73, V9, V27, VI3, VI9 and VI15) and one unaffected member of the family (IV40) were selected for exome sequencing. The whole exome-enriched library was built using NimbleGen SeqCap EZ Exome 64 Mb solution-based SeqCap EZ capture reagents, and solution hybridization exome capture was conducted in according with the manufacturer’s protocol. Exome sequencing was taken by using an Illumina HiSeq2000 sequencer.

### Short-read alignment, variant calling and annotation

Low quality reads and PCR duplicates with >5 unknown bases were eliminated [15], for insertion/deletion (indel) and single nucleotide polymorphism (SNP), respectively. Aligning between read and the National Center for Biotechnology Information human reference genome (hg 19) were performed by sequencing reads were aligned to using Burrows-Wheeler Aligner (BWA) [15] and Short Oligonucleotide Analysis Package (SOAP3) tools [16]. Indels were validated according to the alignment result with the Genome Analysis Toolkit (GATK), and SNP calling was performed with Short Oligonucleotide Analysis Package (SOAPsnp). Variants were annotated using ANNOVAR tool.

### Validation of mutation by Sanger sequencing

Sanger sequencing was used to validate the variants identified by exome sequencing. Specific primers were designed by Primer Premier 3.0 software for the target region. Genomic DNA from participants and 100 normal controls was analyzed.

Genomic DNA samples were amplified with the forward primer (5′- GCAGATCATCTTCGTCTCCA-3′) and the reverse primer(5′- GGCCACAGACAACATGAACA-3′). The following program was used: 95 °C for 3 min (1 cycle); 95 °C for 30 s, 60 °C for 30 s, 72 °C for 30 s (30 cycles); 72 °C for 10 min (1 cycle).

### Bioinformatics analysis

The effects of wild-type amino acid sequences with the p. D47N mutant of *Cx50* on the secondary structure were performed using Antheprot 2000 software (version 6.6.5, IBCP, Lypn, France). The solved structure of gap junction protein beta 2(Cx26) was taken as template (Protein Data Bank No.2ZW3). The model structure of homomeric wild-type and the mutant of GJA8 were modelled by Swiss-Model Server [17]. In addition, the possible functional effect of the amino acid change was predicted by PolyPhen-2 and SIFT.

## Results

### Clinical evaluations

Among 171 members in this six-generation Chinese family, affected individuals account for 23.39% (Fig. 1). All affect individuals in the pedigree had bilateral cataracts. Autosomal dominant inheritance mode of the congenital cataract was ascertained by the presence of affected individuals in each generation of the family, and male-to-male transmission. The proband’s son (VI 9) had been diagnosed with cataracts when he was 15 months old. Slit-lamp examination of his left eye showed perinuclear cataract.

### Identification of Cx50 mutation

Whole exome sequencing was performed on genomic DNA from nine patients of congenital cataract family (III12, IV11, IV28, IV30, IV73, V9, V27, VI3, VI9 and VI15) and one unaffected individual (IV40) though next-generation sequencing technology. As demonstrated in Table 1, we obtained at least 64.06 million reads that mapped to targeted exome regions; more than 99.49% of the target region was covered. The mean depth of the target exome region was 180.98×, 191.56×, 191.23×, 155.43×, 184.67×, 197.75×, 203.48×, 160.48×, 167.92×, 155.12× and 187.92×, respectively. The raw Indel/SNP sequencing data are shown in Table 2. To help identify candidate mutations, untranslated regions, variants falling within intergenic, synonymous substitutions, intronic were excluded. Then the remaining variants were filtered out in at least four public genetic variant databases, including 1000 Genomes, dbSNP, HapMap and YH. Variants with an allele frequency > 0.5% were rejected. Variants shared by 10 patients and absent from 1 unaffected individual were analyzed.

**Table 1:** Coverage statistics with next-generation sequencing in ten patients with autosomal dominant congenital cataract and one unaffected member of family

Sample	III12	IV11	IV28	IV30	IV73	V9	V27	VI3	VI9	VI15	IV40
Total base mapped (G)	11.6	12.31	12.15	9.72	11.92	12.35	13.16	9.65	10.37	11.51	11.84
Region of target kit	64,558,893	64,326,610	64,326,610	64,326,610	64,558,893	64,558,893	64,558,893	64,326,610	64,326,610	64,326,610	64,326,610
Region of covered on target	64,226,731	64,090,887	64,126,819	64,109,790	64,332,815	64,368,416	64,322,956	64,060,274	64,112,774	64,117,790	64,141,226
Coverage of target region (%)	99.49	99.63	99.69	99.66	99.65	99.7	99.63	99.59	99.67	99.68	99.71
Effective bases on target (G)	11.68	12.32	12.3	10	11.92	12.77	13.14	10.32	10.8	9.98	12.09
Average sequencing depth on target region	180.98	191.56	191.23	155.43	184.67	197.75	203.48	160.48	167.92	155.12	187.92
Target coverage with at least 5× (%)	98.72	98.93	99.02	98.93	98.98	99.11	99.01	98.78	98.96	98.92	99.05
Target coverage with at least 10× (%)	98.07	98.32	98.42	98.19	98.37	98.54	98.47	98.09	98.32	98.24	98.45
Target coverage with at least 20× (%)	96.99	97.25	97.31	96.57	97.21	97.42	97.54	96.83	97.17	96.92	97.42
Flank region coverage with at least 5× (%)	22.26	18.67	18.83	18	18.1	17.42	17.67	17.1	16.92	17.07	16.61
Flank region coverage with at least 10× (%)	17.82	14.06	13.87	13.12	13.48	12.06	12.01	13.14	13.22	11.6	11.37
Flank region coverage with at least 20× (%)	15.16	11.79	11.58	10.7	11.18	9.94	9.88	10.88	11.02	9.34	9.34
Exome coverage with at least 5× (%)	98.1	98.3	98.4	98.3	98.4	98.6	98.4	98	98.3	98.3	98.4
Exome coverage with at least 5× (%)	97.2	97.4	97.6	97.4	97.6	97.8	97.6	97	97.4	97.3	97.5
Exome coverage with at least 5× (%)	95.9	96.2	96.4	95.9	96.4	96.6	96.5	95.6	96.1	95.9	96.3

**Table 2:** Variations identified by whole exome sequencing

Mutation type	III12	IV11	IV28	IV30	IV73	V9	V27	VI3	VI9	VI15	IV40
Indel analysis											
Total	15,930	15,690	15,489	13,623	15,448	15,678	18,613	14,028	14,873	14,520	15,657
1000genome and dbsnp	6813	6707	6678	6153	6663	6561	8340	6123	6324	6375	6586
1000genome specific	151	134	128	132	131	143	170	154	129	128	122
dbSNP specific	4846	4570	4462	3853	4544	4448	5594	4036	4406	4324	4559
dbSNP rate	73.19%	71.87%	71.92%	73.45%	72.55%	70.22%	74.86%	72.42%	72.14%	73.68%	71.18%
Novel	4120	4279	4221	3485	4110	4526	4509	3715	4014	3693	4390
Homozygous	4857	4935	4803	4405	4612	4448	3181	4304	4534	4601	4686
Heterozygous	11,073	10,755	10,686	9218	10,836	11,230	15,432	9724	10,339	9919	10,971
Frameshift	374	413	394	394	406	458	423	392	417	387	397
Non-frameshift Insertion	158	180	189	153	181	208	195	173	189	164	169
Non-frameshift Deletion	61	62	63	67	66	81	83	68	66	72	60
Non-frameshift codon substitution plus Insertion	61	77	61	58	73	80	88	70	75	55	84
Non-frameshift codon substitution plus Deletion	28	28	35	25	33	34	30	38	25	23	26
Stopgain	4	14	10	4	9	5	7	9	9	10	12
Stoploss	1	1	0	0	1	2	2	1	1	1	1
Startloss	0	1	0	0	2	0	2	1	0	1	1
Exonic	689	777	754	702	772	869	832	754	782	715	751
Splicing	62	58	57	59	62	60	60	63	61	57	66
NcRNA	238	229	240	235	233	249	259	239	223	222	248
UTR5	178	194	188	180	186	198	216	174	172	174	203
UTR3	1530	1510	1427	1276	1498	1471	1797	1372	1414	1382	1519
Intronic	11,915	11,636	11,562	10,061	11,403	11,572	13,936	10,245	10,997	10,788	11,579
Upstream	283	280	307	266	304	293	338	242	279	239	284
Downstream	733	710	683	603	740	708	846	663	682	688	738
Intergenic	302	296	271	241	250	258	329	276	263	255	269
SNP analysis											
Total	134,311	134,225	136,378	129,878	134,039	133,761	166,869	127,698	130,216	131,224	134,002
1000genome and dbsnp	121,404	120,889	122,334	116,489	120,656	119,805	152,022	114,903	117,222	117,467	119,890
1000genome specific	443	456	451	450	466	500	503	473	473	443	436
dbSNP specific	4979	5030	5142	4963	5008	5188	5533	4875	5051	5034	5008
dbSNP rate	94.10%	93.81%	93.47%	93.51%	93.75%	93.45%	94.42%	93.80%	93.90%	93.35%	93.21%
Novel	7485	7850	8451	7976	7909	8268	8811	7447	7470	8280	8668
Homozygous	51,982	53,569	52,947	51,462	51,793	50,013	32,944	48,638	50,529	51,424	51,815
Heterozygous	82,329	80,656	83,431	78,416	82,246	83,748	133,925	79,060	79,687	79,800	82,187
Synonymous	11,043	11,075	11,209	10,961	10,967	11,123	14,116	11,169	11,048	11,104	11,215
Missense	10,750	10,857	10,991	10,820	10,768	11,029	13,713	10,878	10,892	10,768	10,992
Stopgain	100	113	117	110	102	109	139	113	111	117	113
Stoploss	30	33	31	30	28	35	34	33	31	35	27
Startgain	506	509	496	468	524	491	639	466	487	495	503
Startloss	30	29	26	27	30	30	36	24	29	30	32
Exonic	21,979	22,126	22,390	21,970	21,916	22,346	28,067	22,241	22,131	22,075	22,402
Splicing	159	162	166	149	150	156	191	168	157	157	158
NcRNA	3252	3279	3410	3233	3381	3329	3809	3283	3281	3245	3284
UTR5	1981	2028	2080	1993	2015	2096	2498	1908	1948	1968	2061
UTR3	7707	7707	7821	7485	7825	7652	9778	7461	7527	7610	7820
Intronic	89,844	89,674	91,108	86,065	89,445	88,742	111,479	83,963	86,127	87,172	89,093
Upstream	2248	2299	2339	2195	2262	2344	2743	2040	2170	2152	2237
Downstream	4596	4483	4540	4325	4523	4471	5408	4202	4364	4429	4516
Intergenic	2545	2467	2524	2463	2522	2625	2896	2432	2511	2416	2431
SIFT	1859	1905	1934	1819	1833	1939	2556	1918	1866	1892	1904

**Fig. 2:**
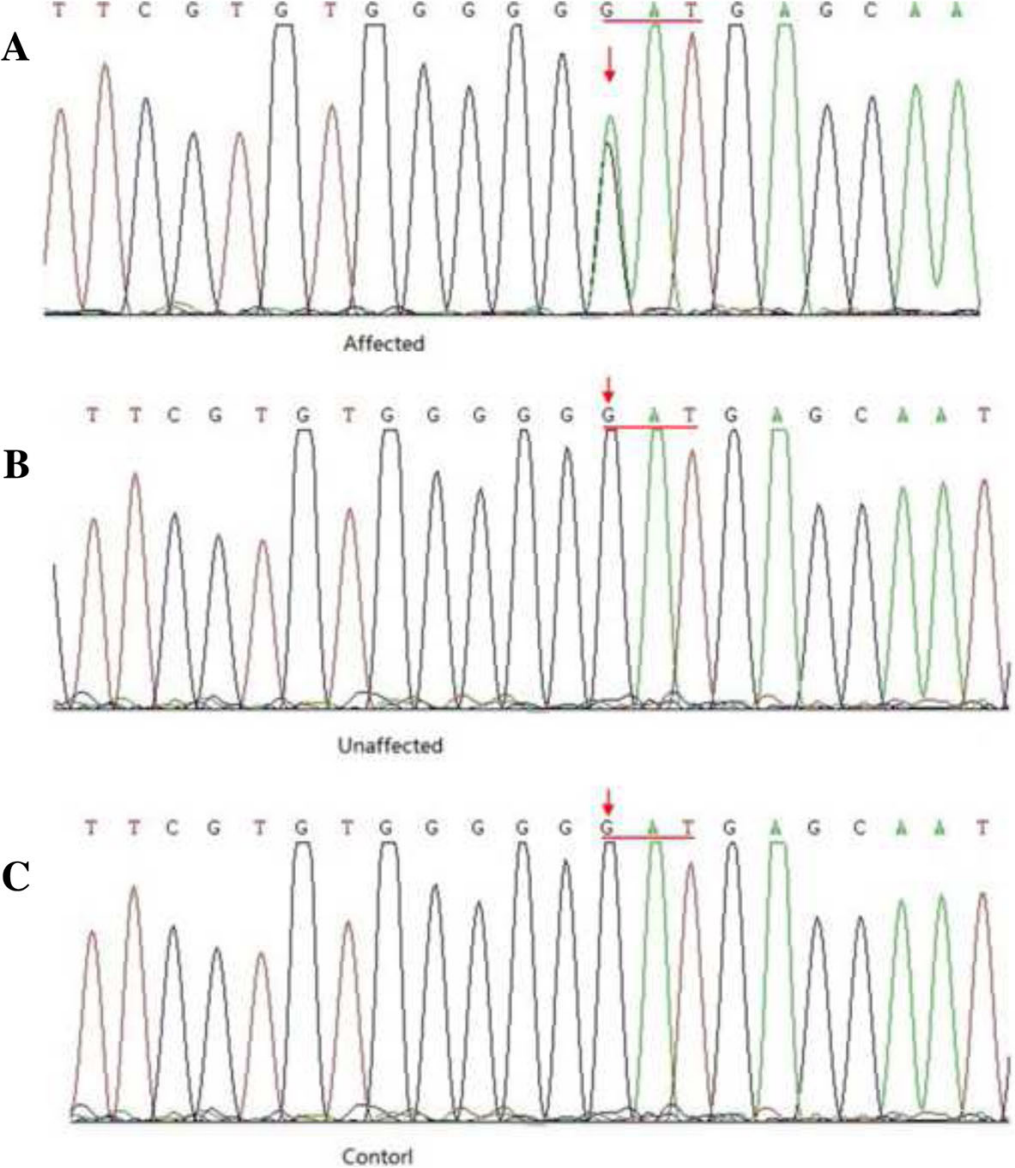
The mutation in *Cx50* was confirmed with Sanger sequencing. **a** a heterozygous mutation c.139 G > A was identified in all affected participants. **b** Sequence of unaffected individual. **c** Sequence of control. The amino acid reading-frame is indicated, GAT encodes Asp (D), and AAT encodes Asn (N)

After filtering and samples comparison, one heterozygous change was confirmed in all affected individuals in congenital cataract family, G > A, at position 139 (c.139 G > A) in exon 2 of GJA8 (Cx50). This change led to the substitution of aspartic acid by asparagine at position 47 (p. D47N). This mutation was further confirmed by Sanger sequencing (Fig. 2). The D47N substitution co-segregated with all 14 affected individuals, while it was not found in the unaffected family members or in the 100 healthy controls.

### Bioinformatics analysis

**Fig. 3:**
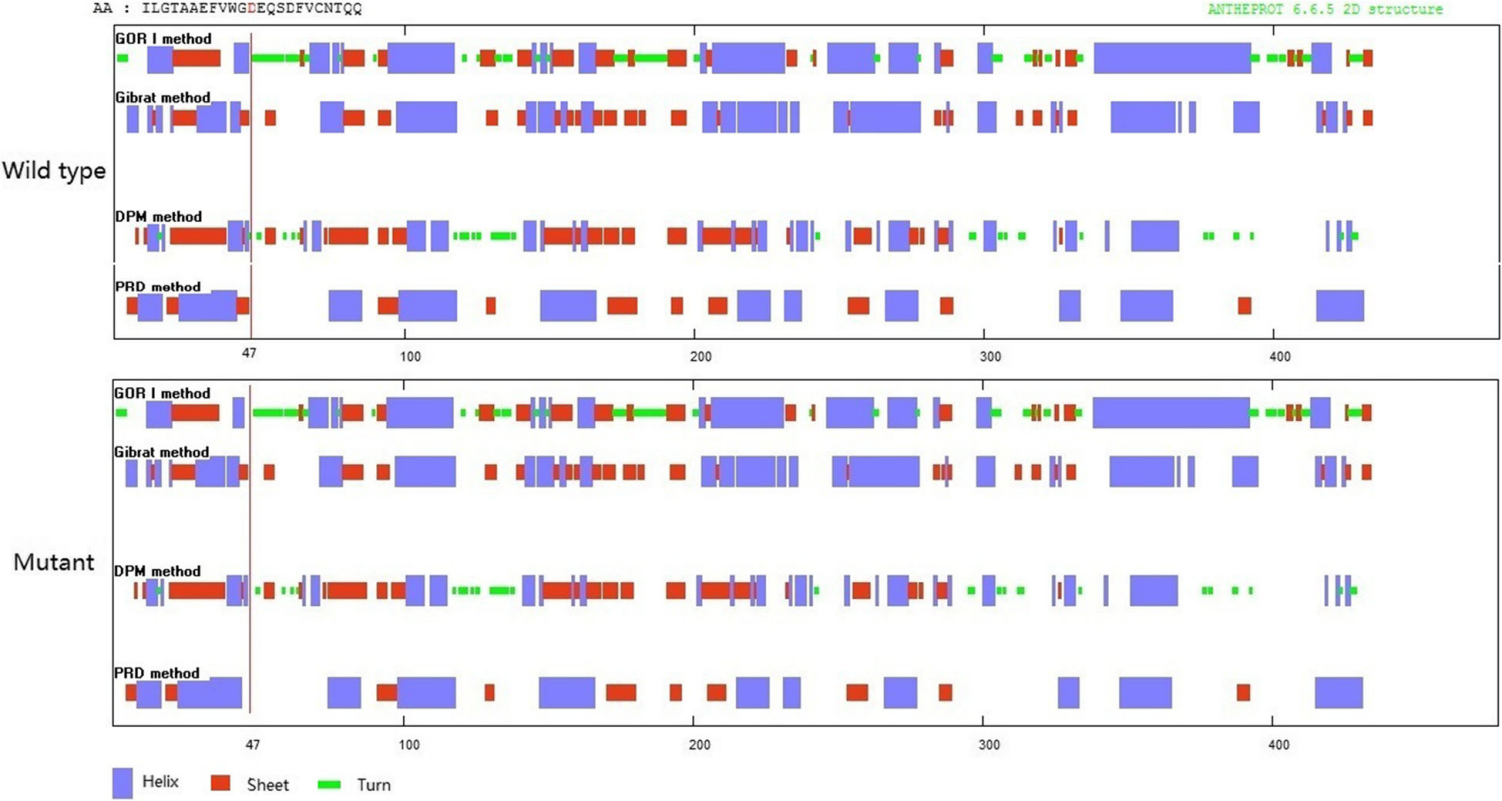
Comparison of the secondary structure of wild type and mutant. The *red line* indicates the position of 139 G > A

**Fig. 4:**
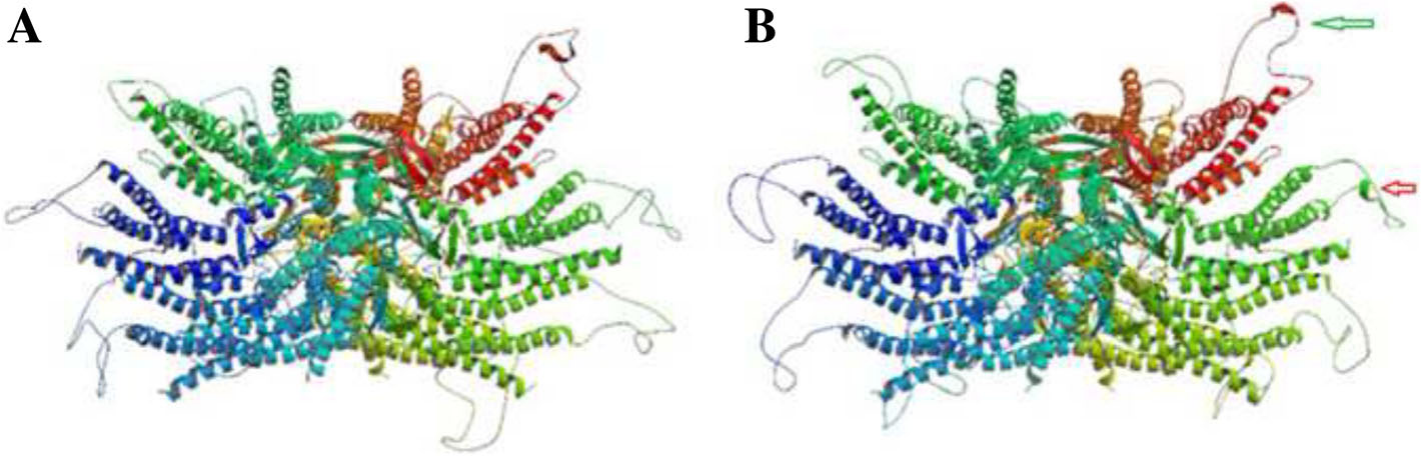
Structure homology modeling and comparison of muant protein and wild type Cx 50*.* **a** Wild type Cx50*.* **b** Mutant protein Cx50

The potential structure and function impact of the D47N mutation was predicted to affect protein function with a score of 0.00, and could probably be damaging with a score of 1.0 by SIFT and PolyPhen-2, respectively. As shown in Fig. 3, the secondary structure of mutant Cx50 protein was different with wild type. The results stated clearly that the wild-type sheet in COOH- terminal portion is likely missing in the D47N mutant. Took the structure of Cx-26 as template, the model structure of the mutant Cx50 have distinct changes (Fig. 4). There are additional helix (red arrow) and shortened sheet (green arrow) in the D47N mutant.

## Discussion

In the current study, we confirmed a missense mutation c. 139 G > A in Cx50 (GJA8) in a six-generation Chinese pedigree with congenital cataract. This mutation resulted in an asparagine substitution for aspartic at amino acid residue 47 (D47N).

Cataracts are defined as opacification of the normally transparent crystalline lens, and are the leading cause of vision loss in the world. Congenital cataract is a type of cataract that emerges at birth or during early childhood [5, 18]. The abnormality of lens can interfere with normal development of eyes [5, 19]. Congenital cataracts can be inherited or familial, either as an isolated lens phenotype or as part of a genetic/metabolic disorder, commonly with full penetrance and autosomal dominant transmission [19]. Genetic factors play an important role in congenital cataract [20]. Gene mutations that affecting the lens development during embryonic period are considered to be the main cause [18]. Up to now, more than 39 genes and loci have been confirmed to be involved in the formation of isolate cataract [21, 22], including crystallins, such as α-, β-, γ-crystallins (e.g., *CRYAA*, *CRYBB1*, *CRYBB2*, *CRYGD*), membrane transport and channel proteins, such as α-connexins (*GJA3*, *GJA8*).

Intercellular gap junction channels provide pathways for metabolic and electrical coupling between cells in different tissues, and they are permeable to ions and small solutes, such as ions (K+, Ca2+), nutrients and small metabolites [23]. Gap junction channels consist of connexin protein subunits. Connexin proteins also known as gap junction proteins have four transmembrane domins with two extracellular loops (E1 and E2) and three intracellular regions (the NH2-terminus, a cytoplasmic loop and the COOH-terminus) [24]. Three isoforms of the connexin gene family- *Cx43* (GJA1), *Cx46* (GJA3) and *Cx50* (GJA8) are abundantly expressed in the vertebrate lens.

*Cx50* is an important protein and play an important role during lens growth, maturation of lens fiber cells, and lens transparency [25]. *Cx50* comprises two exons with exon-2 coding for the entire 433 amino acid residues of gap junction protein α8 (GJA8). Up to date, at least 32 mutations in *Cx50* have been identified to contribute to cataract. Of the 32 coding mutations, 29 result in missense substitutions that are involved in autosomal dominant cataract, and two are frameshift mutation associated with autosomal recessive cataract [6]. The majority of missense substitution are situated in the N-terminal half of the protein, which also contains the conserved connexin domain (amino acids 3–109) [6]. Three types of mutation: D47N, D47H and D47Y indicate that the amino acid at position 47 in GJA8 is a mutational hot spot [26–28]. Functional findings showed that D47N mutant expressed in *Xenopus* oocyte pairs could not form functional gap junction channels. Moreover, co-expression of Cx50D47N with wild-type Cx50 did not inhibit the activity of wild-type Cx50 [29]. The similar behavior was also observed in the mouse Cx50D47A, a mutation underlying the cataracts in the No2 mouse [30]. D47N and D47A mutants were loss-of-function mutants. Cellular level studies showed that the mutation of Cx50 prevented its localization to the plasma membrane. And this may lead to a capacity deficiency of Connexin 50, triggering a complex sequence of events, such as disruption of transmembrane ion gradients, loss of membrane potential, decreased cell growth and subsequent decreased metabolic activity [25, 31]. Cx50 is critical for ball-and-socket structures, actin distribution and fiber cell morphology. Cx50 gap junctional communication through ball-and-socket is important for lens development, especially during rapid, early fiber cell growth [32].

Some limitations of this study should be addressed. First, we did not collect all of pedigree samples, especially the affected individuals in the congenital cataract family. Secondly, we did not perform more experiments, such as cell function experiment of D47N mutant and animal model experiments. Both of these limit our knowledge of more information of the D47N mutant. Nonetheless, advantages in our study should also be acknowledged. Exome sequencing and next-generation sequencing provide a rational approach to screen all candidate genes for inherited cataract or other inherited disease. In addition, exome sequencing and next-generation sequencing are suitable for molecular diagnosis of hereditary diseases. Our finding supports the enormous potential of exome sequencing in molecular diagnosis of single gene disease.

## Conclusions

In conclusion, the present research confirmed a recurrent mutation, c.139 G > A (p.D47N) in *Cx50* in a six-generation Chinese family with autosomal dominant congenital cataract. This result provided further evidence for *Cx50* in association with congenital cataract, and the amino acid at position 47 is a mutational hot-spot. The function of D47N mutation needs to be further certificated in animal mode. In addition, exome sequencing and next-generation sequencing are suitable for molecular diagnosis of hereditary diseases.

## Abbreviations

*CRYAA*: Crystallin Alpha A
*CRYAB*: Crystallin Alpha B
*CRYBA1*: Crystallin Beta A1
*CRYBA3*: Crystallin Beta A3
*CRYBA4*: Crystallin Beta A4
*CRYBB1*: Crystallin Beta B1
*CRYBB2*: Crystallin Beta B2
*CRYBB3*: Crystallin Beta B3
*CRYGA*: Crystallin Gamma A
*CRYGC*: Crystallin Gamma C
*CRYGD*: Crystallin Gamma D
*CRYGS*: Crystallin Gamma S
*Cx43*: Connexin43
*Cx46*: Connexin46
*Cx50*: Connexin50
GJA1: Gap Junction Protein Alpha 1
GJA3: Gap Junction Protein Alpha 3
GJA8: Gap Junction Protein Alpha 8
